# The relationship between resilience and empowering leader behaviour of nurse managers in the mining healthcare sector

**DOI:** 10.4102/curationis.v41i1.1775

**Published:** 2018-06-28

**Authors:** Babalwa Tau, Emmerentia du Plessis, Daleen Koen, Suria Ellis

**Affiliations:** 1 School of Nursing Science, North-West University, Potchefstroom, South Africa; 2 School of Nursing Science, North-West University, Mafikeng, South Africa; 3 Unit for Business, Mathematics and Informatics, North-West University, Potchefstroom, South Africa

## Abstract

**Background:**

The South African mining healthcare sector faces injuries, illnesses including HIV and AIDS and high staff turnover rates. In this sector, nurse managers should create an optimal environment for providing nursing care by motivating, influencing and empowering nurses.

**Objectives:**

This study aimed to investigate the relationship between nurse managers’ resilience and empowering leader behaviour in this sector.

**Method:**

The study employed a quantitative, descriptive and correlational design. The research population comprised 31 nurse managers, 101 professional nurses, 79 enrolled nurses and 79 enrolled nursing auxiliaries who participated in the study. Two questionnaires were used as data collection methods, namely Wagnild and Young’s Resilience Scale Questionnaire to investigate the resilience of nurse managers and the Empowering Leadership Questionnaire to measure empowering leader behaviour of the nurses supervised by a particular nurse manager.

**Results:**

Out of 31 nurse managers, 8 had a low level, 19 had a moderate level and 4 had a high level of resilience. According to Hoteling’s *t*-test the nurse managers in the low resilience group displayed lower empowering leader behaviour as perceived by their team members than those in the high resilience group in terms of the five factors included in the Empowerment Leadership Questionnaire.

**Conclusion:**

Respondents with high resilience scores tended to have higher leader empowering behaviour.

Recommendations include the strengthening of nurse managers’ resilience through workshops and reflection practices, debriefing and performance feedback sessions.

## Introduction

According to Nguyen et al. (2016:13) resilience is positioned in correlation to adversity – to demonstrate resilience, one must first encounter adversity, such as being a nurse leader in the mining healthcare sector required to cope with challenges. Allison (2012:79) in her research stated that in the presence of change and crisis, the resource needed most is resilience.

The question of whether resilience is associated with empowering leadership behaviour is of interest in this working environment. Resilient leadership is crucial in addressing complex issues associated with healthcare system challenges (Allison 2012:79; Jackson & Daly 2011:21).

This article reports on a study that aimed to determine the resilience and empowering leadership behaviour of nurse managers in South Africa’s mining healthcare sector. Understanding the level of resilience of nurse managers and knowing their level of leadership empowering behaviour and the relationship between these two enabled the formulation of a recommendation to strengthen the resilience of nurse managers.

## Background and literature review

Although the gold mining sector brought many economic benefits to South Africa, there were also some social challenges (Stuckler et al. 2013:639). Stuckler et al. described the many challenges that the mining healthcare sector faces, including high levels of occupational injuries, illnesses and non-occupational diseases (such as HIV and AIDS) as well as high staff turnover rates. Moreover, the mining sector is characterised by a high level of occupational diseases such as silicosis and risks such as mine incidents. Mclaggan, Bezuidenhout and Botha (2013:1) stated that the mining industry is especially faced with challenges that include labour unrest, skills shortages, increased demand for productivity and high turnover. This places a great challenge on the mining healthcare sector, which renders services to the mining industry.

Nurses working in the mining healthcare sector have to carry the burden of providing nursing care to mine workers. This, in turn, leads to nurses being overworked and under pressure. These challenges have been associated with problems in retaining a viable nursing workforce (Jackson, Firtko & Edenborough 2007:2).

Positive leadership qualities and strong facilitative leadership behaviour of first-line managers are therefore crucially important in creating an environment that increases job satisfaction and nurses’ intention to stay (Sellgren, Ekvall & Tomson 2008:584). According to Pillay (2011:176), nurse managers should take the lead in addressing challenges hampering the delivery of health services in the mining sector. Effective nurse leaders should be able to address complex issues associated with healthcare system changes to overcome the challenges they face (Bester, Stander & Van Zyl 2015:1; MacPhee et al. 2011:159). Therefore, when facing these challenges, nurse managers in the mining healthcare sector have to display effective leadership skills.

Irrespective of the specific leadership styles and skills used, empowerment and sharing of power are innovative ways of encouraging all healthcare professionals to become involved in and committed to their work situation. Liu (2015:477) stated that empowering leadership behaviour is a series of management practices including participation and information sharing. Banutu-Gomez (2015:342) also stated that the role of leaders is to empower teams to successfully complete necessary leadership functions themselves.

Arnold et al. (2000:265) mentioned five categories of leadership behaviour constructs required for empowering teams, namely: (1) leading by example: as a set of behaviours displaying the commitment of the leader to his or her own work and that of the team, (2) coaching: behaviour that educates team members and assists them to become self-reliant, (3) showing concern: behaviour that shows a regard for team members’ wellness, (4) informing: when the leader includes the team members in information pertaining to the vision and mission of the company and (5) participative decision-making: team members’ inputs and information are considered by the leader, and team members are encouraged to express their opinions and ideas.

Similarly Kotzè et al. (2008:47) stated that effective leadership is a process of interaction where a leader influences others towards achieving a goal. Arnold et al. (2000:265) state that empowered teams have more autonomy, self-direction and control over their working environment. This produces job satisfaction, cost-effectiveness, better solutions and constructive conflict management (Maboko 2011:912). Effective leadership in adverse conditions – such as in the mining healthcare sector – can therefore be defined as:

competencies and processes required to enable and empower ordinary people to do extraordinary things in the face of adversity, and constantly turn into superior performance to the benefit of themselves. (Meyer et al. 2011:208)

Nurses’ attitudes and work performance are affected by the leadership behaviour of nurse managers. Bester et al. (2015:11) agreed, stating that employees’ perception of their leaders’ empowering behaviour predicts intention to leave the organisation. It is therefore clear that nurse managers must develop empowering leadership skills, resilience, healthy relationships and conflict management skills while achieving productive goals. Moreover, given the high level of pressure, uncertainty and rapid changes accompanied by the challenges in the mining healthcare system it is evident that nurse managers need to be resilient (Rivers et al. 2011:48). According to Jackson and Daly (2011:21), resilient leaders not only have the ability to survive in difficulty and adversity but are able to display behaviour that will enhance subordinates’ ability to thrive. They furthermore questioned the fact that concepts like adversity and resilience are seldom discussed in relation to nurse leaders, yet nurse leaders work in a context of great workplace difficulty.

Research linking resilience and leadership can be found (Harland et al. 2005:4; Nguyen et al. 2016:14). Harland et al. (2005:4) theorised on a link between leadership and resilience. These authors stated that developing the capacity for resilience is a vital component of effective leadership. Shahrazad et al. (2012:64) found significant correlations between leadership and resilience, namely the higher the skills of leadership, the higher the ability to be resilient and to overcome challenges.

## Problem statement

Nguyen et al. (2016:13) in their study confirmed the findings of Harland et al. (2005:4) that the application of leadership to resilience has been largely ignored, and they suggested that such research should be undertaken. There seems to be a link between leadership and resilience, but currently the resilience and empowering leadership behaviour of nurse managers in the mining healthcare sector has not been explored. The fact that some nurse managers in the mining healthcare sector are able to cope with unexpected setbacks and overcome adversities and display effective leadership, as well as the fact that there might be a relationship between resilience and empowering leader behaviour (Shahrazad et al. 2012:64), prompted this investigation about the relationship between resilience and empowering leadership behaviour of nurse managers in the mining healthcare sector. The researchers were therefore interested in understanding how some nurse managers, as leaders, are able to overcome adversities and turn them into developmental experiences through empowering leadership behaviour.

## Research objectives

The objectives of this study were to:

- Determine the resilience of nurse managers in the mining healthcare sector.
- Determine the empowering leader behaviour of nurse managers in the mining healthcare sector as perceived by nurses working in teams with these managers.
- Determine the relationship between resilience and empowering leader behaviour of nurse managers in the mining healthcare sector.

## Definition of key concepts

### Resilience

*Resilience* is the personal qualities and skills that allow for an individual’s healthy and successful functioning or adaptation within the context of significant adversity or a disruptive life event (Lee et al. 2013:269). According to Fiksel (2014:2) personal resilience is being resourceful and having a strong character. In this study *resilience* refers to the ability of the nurse managers in the mining healthcare sector to deal with adversity in the workplace.

### Empowering leader behaviour

According to Hon and Chan (2013:199), empowering leader behaviour includes leading by example, coaching, showing concern, informing and participative decision-making. In this study *empowering leader behaviour* refers to the sharing of power of nurse managers with the view of enhancing employees’ motivation and investment in their work

### Nurse manager

A nurse manager is a nurse leader for a specific unit or area. In the mining healthcare sector the nurse manager manages the budget, medical records, employee performance evaluation, staff recruitment and retention, and he or she is also responsible for safety programmes.

### Mining healthcare sector

A mining healthcare sector includes hospitals, primary healthcare clinics, biotechnology and a variety of medical products. The mining healthcare sector provides healthcare to people working in the mining industry and their families. This research focused on the mining healthcare sector in the North West and Gauteng provinces of South Africa.

### Contribution to the field

Findings from this study inform us about the level of resilience of nurse managers and their level of leader empowering behaviour. Knowing the level of resilience of nurse managers enables us to strengthen the resilience of nurse managers and enables deeper probing into the concepts of resilience and leader empowering behaviour.

## Method

### Research design

A quantitative, descriptive and correlational design was used in this study.

### Context of the study

This research was conducted in the South African operations of a mining group consisting of two hospitals, eight medical stations (also referred to as primary healthcare [PHC] clinics) and two occupational health centres. These healthcare facilities are situated in the North West and Gauteng provinces.

### Population and sampling

In this research two groups were included. The population included 31 nurse managers as one group and 259 nurses working in teams with these nurse managers as the other group.

The first group comprised 31 nurse managers. The second group comprised professional nurses (*n* = 101), enrolled nurses (*n* = 79) and enrolled nurse auxiliaries (*n* = 79) registered and enrolled with South African Nursing Council working in the teams of the participating nurse managers.

An all-inclusive sampling technique was ideal because all members of these groups working in the specific mining healthcare sector where the research took place could be included.

### Research instruments

The instruments used for data collection were self-administered questionnaires, which means that the respondents completed the instruments themselves (Polit & Beck 2008:414). This study utilised two questionnaires as data collection methods, namely the Resilience Scale (RS) developed by Wagnild and Young (1993) and reviewed by Wagnild (2009) and the Empowering Leadership Questionnaire (ELQ) developed by Arnold et al. (2000).

The RS was completed by nurse managers (*n* = 31) and was used to collect data to determine their resilience. The questionnaire consists of 25 statements using a Likert scale ranging from 1 (strongly disagree) to 7 (strongly agree). The possible scores for the RS range from 25 to 175. Scores greater than 160 indicated high resilience, scores between 145 and 160 indicated moderately high to high resilience levels, scores from 121 to 145 indicated moderately low to moderate resilience levels, and scores of 120 and below indicated low resilience levels. A Cronbach’s alpha coefficient of 0.95 was obtained for this study.

The ELQ was used to collect data that determined the empowering leader behaviour of nurse managers in the mining healthcare sector. The questionnaire was completed by 259 nurses, who were supervised by the specific nurse managers who completed the RS. The questionnaire measures five factors of leadership behaviour for empowered teams. The five categories are leading by example, coaching, participative decision-making, informing, and showing concern and interacting with the team (Arnold et al. 2000:249). The questionnaire consists of 38 items. The questionnaire is a five-point response scale, where 1 = ‘never’ and 5 = ‘always’.

### Data collection method

Data for the study were collected during April 2015. The mediator, who was the health service manager for both healthcare facilities where data were collected, distributed questionnaires to 31 nurse managers and obtained informed consent. An information sheet describing the details of the study was also handed to the second group of respondents, nurses working in the teams of the nurse managers. Those who gave consent were then handed questionnaires. In order to ensure confidentiality, the respondents were requested not to include their names or any form of identification on the questionnaires. Respondents were allowed to complete the questionnaires at their own convenience.

### Data analysis

Descriptive and inferential statistics were utilised to convert and condense the data into organised representations. The Statistical Package for the Social Sciences (SPSS) version 22 (2011) statistical program was used for data analysis and the guidelines from the ‘Resilience Scale User’s Guide’ were also used (Wagnild 2011:72).

Correlations were calculated between the resilience of nurse managers using the RS questionnaire and the ELQ, taking into account the dependency between nurse managers and team members working with nurse managers. To examine this relationship further, nurse managers were categorised into groups of low, moderate and high resilience according to the guidelines of the developers. A multivariate Hoteling’s *t*-test was performed to determine whether there was a statistical significant difference between the leadership sub-scale scores for nurse managers as perceived by their team members in low and high resilience categories.

### Ethical considerations

Ethical approval was granted by the Health Research Ethics Committee of the North-West University (NWU), Potchefstroom Campus (NWU-00161-14-A1). Permission to access the mining healthcare sector and to conduct the study was obtained from the health service manager of the mining healthcare sector.

## Results

### Demographics

All the nurse managers (*n* = 31) were females and their ages ranged between 38 and 53. The nurse managers were from both hospitals, which are urban hospitals.

Table 1 provides a breakdown of the number of nurse managers and teams working under each nurse manager’s supervision specific units. In both hospitals (in both regions) the numbers were the same.

**TABLE 1:** Nurse managers and their teams in the mining healthcare sector.

Nurse managers	Professional nurses	Enrolled nurses	Enrolled nursing auxiliaries
*n* = 31	*n* = 101	*n* = 79	*n* = 79

Medical stations are smaller than the hospitals; therefore the numbers were lower. On average for each nurse manager there were two (*n* = 2) professional nurses, one (*n* = 1) enrolled nurse and two (*n* = 2) enrolled nursing auxiliaries. Because of loss of confidentiality of respondents, no other demographic details were collected.

### Resilience of nurse managers

The resilience questionnaire included five characteristics that consisted of 25 items. The means of the sub-scale scores of the RS are shown in Table 2. The first characteristic is purposeful life (meaning), the realisation that life has a purpose and the sense of having something for which to live; it had a mean of 28.2, which was the highest sub-score. Perseverance is the act of persistence despite adversity or discouragement, showing willingness to continue to reconstruct one’s life and remain involved; it had a mean of 27.8. Self-reliance is a belief in oneself and in one’s capabilities; it had a mean of 28.1.

**TABLE 2:** Resilience scale: Maximum and minimum scores, mean and standard deviation of the respondents

Variable	Cronbach’s alpha	Minimum	Maximum	Mean	Standard deviation
Resilience	0.953	88.00	168.00	138.53	21.16
Purposeful life	0.803	19.00	34.00	28.17	4.26
Perseverance	0.778	15.00	35.00	27.77	4.80
Self-reliance	0.805	17.00	35.00	28.06	4.33
Existential aloneness	0.826	12.00	35.00	26.77	6.05
Equanimity	0.648	18.00	32.00	27.78	3.96

Existential aloneness, the realisation that a person’s life path is unique, with a mean of 26.8, had the lowest sub-score. Equanimity is a balanced perspective of one’s life and experiences and it had a mean of 27.8.

The average resilience score of all respondents was 138.5, indicating that this sample of nurse managers had a moderate level of resilience (see Table 2).

The results indicated that 25.8% (*n* = 8) of the nurse managers had a *low* level of resilience of 120 or lower, 61.3% (*n* = 19) had a moderate level of resilience, and 12.9% (*n* = 4) had a high level of resilience, above 160 (see Table 3).

**TABLE 3:** Summary of managers and nurse respondents in the managers’ team, categorised by level of resilience of the manager.

Level of resilience	Managers		Nurses in managers’ team	
	Frequency	Percentage	Frequency	Percentage
Low (120 or below)	8	25.8	49	18.9
Medium	19	61.3	173	66.6
High (above 160)	4	12.9	37	14.3
**Total**	**31**	**100.0**	**259**	**100.0**

### Empowering leader behaviour of nurse managers

The mean scores of the ELQ are shown in Table 4, where the average of all items contributing to the score was used to determine the sub-scale scores. This implies that the means can be interpreted on the original five-point Likert scale of measurement.

**TABLE 4:** Reliability, mean and standard deviation of the Empowering Leadership Questionnaire.

Variables	Cronbach’s alpha	Minimum	Maximum	Mean	Standard deviation
Leading by example	0.935	1.00	5.00	3.90	0.98
Participative decision-making	0.833	1.00	5.00	3.49	0.87
Coaching	0.964	1.00	5.00	3.71	0.98
Informing	0.930	1.00	5.00	3.95	0.85
Show concern	0.963	1.00	5.00	3.61	1.03

The mean for ELQ scale for *leading by example*, which had five items, was 3.89 (SD 0.982), indicating that managers are committed to their work as well as the work of team members. The mean of *participative decision-making*, which had six items, was 3.48 (SD 0.869), indicating that team members are of the opinion that managers are involving teams in decision-making. The mean for *coaching*, which loaded 11 items, was 3.70 (SD 0.977). This indicated that managers displayed behaviours that educated team members and helped them to become self-reliant. The mean for *informing*, which had six items, was 3.95 (SD 0.854), which was the highest and this indicated that the managers disseminated information such as mission and philosophy as well as other important information pertaining to the company. The mean for *showing concern*, which had 10 items, was 3.60 (SD 1.029), which indicated that managers demonstrated a general regard for team members’ well-being (see Table 4). All these sub-scale scores were positive, with means larger than 3.5.

### Relationship between resilience and empowering leader behaviour of nurse managers

Three approaches were used to test the relationship between resilience and empowering leader behaviour. In the first approach, a correlation matrix was used to test the magnitude, direction and strength of the relationship between the variables. In the second approach, the univariate *t*-tests of independent groups was used to test the statistical significance between the means of each category of leader empowering behaviour as perceived by team members between high and low resilience managers. Thereafter a multivariate Hoteling’s *t*-test was performed to determine whether there was a statistical significant difference between the leadership sub-scale scores for nurse managers as perceived by their team members in low and high resilience categories.

### Correlation results

The correlation matrix representing the empowering leadership factors of the ELQ in relation to resilience is given in Table 5. No statistical or practically significant correlations of ELQ with resilience were found.

**TABLE 5:** Results of correlation between resilience and Empowering Leadership Questionnaire (ELQ) as well as Hoteling’s *t*-test on ELQ sub-scores for high and low resilience groups.

ELQ sub-factors	Correlation with resilience	Resilience group	Means	Standard deviation
Lead by example	0.006	Low	3.86	0.97
	-	High	4.03	0.94
Participative decision-making	0.055	Low	3.41	0.81
	-	High	3.75	0.87
Coaching	0.038	Low	3.61	1.01
	-	High	3.90	1.06
Informing	0.038	Low	3.84	0.90
	-	High	3.98	0.88
Showing concern	0.041	Low	3.49	1.01
	-	High	3.80	1.04

Note: Wilks’ lambda = 0.077; *p* < 0.001.

### Independent *t*-test results

Seeing that no statistically significant correlations of ELQ with resilience were found when using a correlation matrix, to investigate this relationship further the *t*-test was used. The independent *t*-test was conducted between team members with managers in the low and high categories of resilience. This is a procedure used to test the statistical significance of a difference between the means of two groups.

Although the individual tests showed no statistical or practically significant differences between high and low resilience groups, all the ELQ scores in the high resilience group were higher than those in the lower group and a multivariate approach seemed to be viable.

### Hoteling’s *t*-test results

When Hoteling’s *t*-test was performed on the ELQ scores, statistically significant differences between the low and high resilience groups showed statistically significant differences with Pillai’s trace, Wilks’s lambda, Hoteling’s trace and Roy’s largest root, indicating *p* < 0.001. The generalised eta-squared effect size based on lambda for this multivariate test is 1 – lambda = 1 – 0.077 = 0.923, which indicates a large effect according to Steyn and Ellis (2009), where eta-squared of 0.02 is considered as a small effect, 0.13 as a medium effect and 0.26 as a large effect (see Table 5).

### Potential benefits and hazards

The risk anticipated in this study was that managers could feel exposed if team members commented on their behaviour. The researcher therefore ensured anonymity and confidentiality by ensuring that no names would be used, only codes. All questionnaires were completed anonymously. The respondents did not receive any remuneration and there were no direct benefits for them.

### Recruitment procedures

The researcher requested the health service manager to act as mediator. Recruitment was conducted by the health service manager, who was an independent person. The researcher organised a meeting with the permission of the health service manager, who was present at the meeting to explain the purpose of the research.

### Informed consent

The nurse managers and their teams were informed about the purpose and nature of the study. The mediator obtained voluntary informed written consent from the prospective respondents. Respondents were given time to consider the invitation and were informed that they could withdraw at any time without incurring any risk to their well-being. The respondents were not coerced to participate in the study.

### Data protection

Privacy was protected because respondents’ names and information were kept confidential. No names of respondents were used in the research report. Codes were used in the research report to protect the identity of the respondents. The data would be destroyed after a period of seven years.

## Reliability

Cronbach’s alpha coefficients, based on inter-item correlations, were used to determine the reliability of the RS questionnaire. Cronbach’s alpha tests the internal consistency of a measuring instrument (Botma et al. 2010:177). Both internationally and in South Africa the RS has demonstrated reliability, with alpha coefficients ranging from 0.85 to 0.94. The RS was also used in the context of South Africa by Koen, Van Eeden and Wissing (2011:4) using 312 professional nurses employed in public and private hospitals and primary healthcare clinics, and a Cronbach’s alpha coefficient of 0.95 was obtained. The Cronbach’s alpha of the RS was 0.953, which indicated good internal consistency and reliability (see Table 2) (Wagnild 2009:106).

Arnold et al. (2000:249) in their article described the construction and validation of the ELQ. They evaluated the reliability of the ELQ in several organisations. The researchers concluded that the questionnaire indicated satisfactory reliability for all five ELQ sub-scales. The factors of the ELQ scale were divided into five categories of items and had a reliability exceeding 0.80, which also indicated good internal consistency (see Table 4).

## Validity

Validity for both questionnaires was tested. *Validity* referred to the questionnaires’ ability to measure resilience and leadership amongst nurse managers in the mining healthcare sector and implied that the results could be applied to the larger community. The questionnaires were tested for content, face and construct validity.

The RS demonstrated content and construct validity in published studies (Black & Ford-Gilboe 2004; Humphreys 2003; Monteith & Ford-Gilboe 2002; Wagnild 2009). The construction of terms in the RS was those that reflect the generally acceptable definitions of *resilience*. Therefore the instrument was able to investigate the concept of resilience of nurse managers in the mining healthcare sector.

The construction of terms in the ELQ reflected generally acceptable definitions for empowered teams. The validity of the ELQ for this sample was tested by a confirmatory factor analysis, and it yielded satisfactory fit indices with a chi-square value divided by degrees of freedom (CMIN/df) of 2.59, a comparative fit index (CFI) of 0.904 and a root-mean-square error of approximation (RMSEA) of 0.078 with a 90% confidence interval (0.074; 0.083). Because the chi-square test is viewed by some as an overly strict indicator of model fit, given its power to detect even trivial deviations from the proposed model (Hancock & Mueller 2010), Mueller (1996) suggested that CMIN/df be used; interpretation of this value depends to a large extent on the viewpoint of the investigator, but in practice some interpret ratios as high as 3, 4 or even 5 as still representing a good model fit (Mueller 1996). However, it is considered good practice to report multiple fit indices, typically from three broad classes (Hancock & Mueller 2010). Mueller (1996) described values of above 0.9 as indicative of a good overall fit for a CFI. Blunch (2008) stated that models with RMSEA values of 0.10 and larger should not be accepted.

## Discussion

### Resilience

It is evident that this sample of nurse managers on average had a moderate level of resilience. Respondents felt especially proud of their achievements, and indicated that their life had meaning. Areas that might need attention relate to having a purposeful life, existential aloneness and equanimity (see Table 6).

**TABLE 6:** Frequency scores for the Resilience Scale Questionnaire.

Resilience scale items	Strongly agree		Agree		Neutral		Disagree		Strongly disagree		Total	Mean	Standard deviation
	*n*	%	*n*	%	*n*	%	*n*	%	*n*	%			
1. When I make plans, I follow through with them.	17	56.7	8	26.7	4	13.3	1	3.3	0	0.0	30	5.73	1.202
2. I usually manage one way or another.	14	48.3	8	27.7	5	17.2	2	6.9	0	0.0	29	5.38	1.208
3. I am able to depend on myself more than anyone else.	15	48.4	2	6.5	7	22.6	5	16.1	2	6.4	31	5.00	1.770
4. Keeping interested in things is important to me.	20	64.5	6	19.4	4	12.9	1	3.2	0.0	0.0	31	5.68	1.077
5. I can be on my own if I have to.	17	54.9	6	19.4	5	16.1	1	3.2	2	6.4	31	5.32	1.514
6. I feel proud that I have accomplished things in life.	22	71.0	4	12.9	5	16.1	0	0.0	0	0.0	31	6.00	1.125
7. I usually take things in stride.	15	48.7	5	17.2	2	6.9	2	6.9	5	16.1	29	4.83	1.713
8. I am friends with myself.	13	42.0	4	12.9	4	12.9	3	9.7	7	22.7	31	4.68	2.104
9. I feel that I can handle many things at a time.	18	58.1	4	12.9	5	16.1	4	12.9	0	0.0	31	5.29	1.270
10. I am determined.	22	71.0	5	16.1	4	9.7	1	3.2	0	0.0	31	5.94	1.124
11. I seldom wonder what the point of it all is.	9	29.0	9	30.0	7	23.3	4	13.3	1	3.3	30	4.70	1.291
12. I take things one day at a time.	18	60.0	6	20.0	4	13.3	1	3.3	1	3.3	30	5.53	1.408
13. I can get through difficult times because I’ve experienced difficulty before.	20	64.5	8	25.8	3	9.7	0	0.0	0	0.0	31	5.84	0.969
14. I have self-discipline.	19	61.3	3	9.7	6	19.4	2	6.5	1	3.2	31	5.55	1.480
15. I keep interested in things.	20	66.7	6	20.0	4	13.3	0	0.0	0	0.0	30	5.70	0.915
16. I can usually find something to laugh about.	19	61.3	9	29.0	3	9.7	0	0.0	0	0.0	31	5.68	0.871
17. My belief in myself gets me through hard times.	20	66.7	6	20.0	2	6.7	2	6.7	0	0.0	30	5.83	1.206
18. In an emergency, I’m someone people can generally rely on.	23	74.0	6	19.4	0	0.0	2	6.5	0	0.0	31	5.90	1.044
19. I can usually look at a situation in a number of ways.	24	77.5	5	16.1	1	3.2	1	3.2	0	0.0	31	5.87	0.885
20. Sometimes I make myself do things whether I want to or not.	15	48.4	4	12.9	7	22.6	2	6.5	3	9.7	31	4.97	1.622
21. My life has meaning.	23	74.2	3	9.7	4	12.9	1	3.2	0	0.0	31	6.00	1.183
22. I do not dwell on things that I can’t do anything about.	21	67.8	5	16.1	4	12.9	1	3.2	0	0.0	31	5.81	1.138
23. When I’m in a situation, I can usually find my way out of it.	18	58.0	8	25.8	3	9.7	1	3.2	1	3.2	31	5.61	1.283
24. I have enough energy to do what I have to do.	18	57.8	7	22.6	5	16.1	1	3.2	0	0.0	31	5.58	1.119
25. It’s okay if there are people who don’t like me.	22	71.0	4	12.9	5	16.1	0	0.0	0	0.0	31	5.94	1.093

The first characteristic is *purposeful life* (*meaning*), defined by Wagnild and Young (1993) as the realisation that life has a purpose and the sense of having something for which to live. On average respondents in the mining healthcare sector agreed that they seldom wondered what the point of it all was. These results show that respondents felt proud that they had accomplished things in life.

*Perseverance* is the act of persistence despite adversity or discouragement; it shows willingness to continue to reconstruct one’s life and remain involved. According to these results, respondents agreed that *when they made plans, they followed through with them*. It was further evident that most respondents were *determined* and *had self-discipline*. Results showed that the majority of respondents sometimes *made themselves do things whether they wanted to or not*.

*Self-reliance* is a belief in oneself and in one’s capabilities. It is the ability to depend on oneself and recognise limitations and personal strengths. This indicates that respondents agreed that they were able to manage one way or another.

*Existential aloneness* is the realisation that a person’s life path is unique. It confers a feeling of freedom and sense of uniqueness and had the lowest sub-scale score. This finding is an indication that the mining healthcare sector should be aware that some nurse managers might need guidance with regard to existential aloneness, and these respondents therefore may need to strengthen their resilience.

Research has shown that leaders need to be resilient in order to lead their teams. According to Jackson and Daly (2011:21), resilient leaders not only have the ability to survive in difficulty and adversity but are able to display behaviour that will enhance subordinates’ ability to thrive. According to Koen et al. (2011:114), registered nurses show enabling strengths that facilitate resilience in difficult workplace circumstances, and this was also seen with the nurse managers in this research. Moran and Tame (2012:233) confirmed that for organisations to adapt, individuals must work towards a resilient culture. Resilient nurse managers are able to survive and thrive in a context of workplace difficulty, and this will also influence the nurses’ ability to survive and thrive.

### Empowering leadership behaviour

Most nurses felt that nurse managers displayed empowering behaviour, were leading by example and empowered team members through coaching. With regard to participative decision-making, nurse managers used team members’ information and inputs. Furthermore, most nurses felt that the nurse managers encouraged team members to express ideas and that the nurse managers showed concern and cared about the group members’ personal problems and well-being (see Table 7).

**TABLE 7:** Frequency scores for Empowering Leadership Questionnaire (ELQ).

ELQ items	Always		Often		Sometimes		Rarely		Never		Total	Mean	Standard deviation
	*n*	%	*n*	%	*n*	%	*n*	%	*n*	%			
1. Sets high standards for performance by his or her own behaviour.	93	36.3	83	32.4	60	23.2	14	5.4	6	2.3	256	3.95	1.014
2. Works as hard as she or he can.	108	41.9	79	30.5	50	19.4	13	5.0	8	3.1	258	4.03	1.047
3. Works as hard as anyone in my work group.	89	34.8	88	34.4	54	20.8	14	5.5	11	4.3	256	3.90	1.076
4. Sets a good example by the way he or she behaves.	90	35.2	80	30.9	47	18.4	25	9.8	14	5.5	256	3.81	1.178
5. Leads by example.	97	38.0	64	25.1	57	22.4	23	9.0	14	5.5	255	3.81	1.195
6. Helps my work group see areas in which we need more training.	75	29.2	94	36.6	53	20.6	20	7.8	15	5.8	257	3.75	1.131
7. Suggests ways to improve my work’s performance.	71	27.6	95	37.0	55	21.4	22	8.6	14	5.4	257	3.73	1.120
8. Encourages work group members to solve problems together.	79	30.6	79	30.6	54	20.9	33	12.8	13	5.0	258	3.69	1.179
9. Encourages work group members to exchange information with one another.	78	30.2	92	35.7	57	22.1	18	7.0	13	5.0	258	3.79	1.103
10. Provides help to work group members.	69	26.7	103	39.9	51	19.8	24	9.3	11	4.3	258	3.76	1.080
11. Teaches work group members how to solve problems on their own.	61	23.7	101	39.3	57	22.2	24	9.3	14	5.4	257	3.67	1.103
12. Pays attention to my work group’s efforts.	72	28.2	77	30.2	73	28.6	26	10.2	7	2.7	255	3.71	1.069
13. Tells my work group when we perform well.	76	29.6	75	29.2	62	24.1	26	10.1	18	7.0	257	3.64	1.204
14. Supports my work group’s efforts.	65	25.7	87	34.4	56	22.1	30	11.9	15	5.9	253	3.62	1.161
15. Encourages work group members to express ideas and suggestions.	71	27.7	90	35.2	57	22.3	26	10.3	12	4.7	259	3.71	1.118
16. Listens to my work group’s ideas and suggestions.	66	26.0	84	33.1	62	24.4	27	10.6	15	5.9	254	3.63	1.152
17. Uses my work group’s suggestions and makes decisions that affect us.	60	23.3	84	32.7	67	26.1	25	9.7	21	8.2	257	3.53	1.186
18. Gives all work group members a chance to voice their opinions.	83	32.3	73	28.4	58	22.6	22	8.6	21	8.2	257	3.68	1.237
19. Considers my work group’s ideas when she or he disagrees with them.	55	21.4	77	30.0	73	28.4	29	11.3	23	8.9	257	3.44	1.201
20. Makes decisions that are based only on his or her own ideas.	35	13.7	57	22.3	66	25.8	54	21.1	44	17.2	256	2.94	1.293
21. Explains company decisions.	93	36.3	80	31.3	62	24.2	18	7.0	3	1.2	256	3.95	0.997
22. Explains company goals.	102	39.8	83	32.4	53	20.7	14	5.5	4	1.6	256	4.04	0.984
23. Explains how my work group fits into the company.	79	30.9	90	35.2	59	23.0	21	8.2	7	2.7	256	3.83	1.044
24. Explains the purpose of the company’s policies to my work group.	102	39.7	93	36.2	45	17.5	15	5.8	2	0.8	257	4.08	0.934
25. Explains rules and expectations to my work group.	100	38.9	91	35.4	49	19.1	15	5.8	2	0.8	257	4.06	0.940
26. Explains his or her decisions and actions to my work group.	68	26.6	94	36.7	64	24.7	20	7.8	10	3.9	256	3.74	1.057
27. Cares about work group members’ personal problems.	69	27.0	66	25.8	72	28.1	34	13.3	15	5.9	256	3.55	1.187
28. Shows concern for work group members’ well-being.	62	23.9	91	35.5	56	21.9	32	12.5	15	5.9	256	3.60	1.154
29. Treats work group members as equals.	70	27.5	62	24.3	56	22.0	33	12.9	34	13.3	255	3.40	1.362
30. Takes the time to discuss work group members’ concerns patiently.	62	24.6	87	34.5	59	23.4	31	12.3	13	5.2	252	3.61	1.136
31. Shows concern for work group members’ success.	71	27.6	84	32.7	60	23.3	28	10.9	14	5.4	257	3.66	1.152
32. Stays in touch with my work group.	73	28.6	77	30.2	67	26.3	20	7.8	18	7.1	255	3.65	1.177
33. Gets along with my work group members.	65	25.5	84	32.9	70	27.5	22	8.6	14	5.5	255	3.64	1.116
34. Gives work group members honest and fair answers.	72	28.2	73	28.6	67	25.9	26	10.2	17	6.7	255	3.62	1.188
35. Knows what work is being done in my work group.	76	29.7	78	30.5	67	26.2	21	8.2	14	5.5	256	3.71	1.139
36. Finds time to chat with work group members.	81	32.3	73	29.1	56	22.3	20	8.0	21	8.4	251	3.69	1.236
37. Helps my work group focus on goals.	72	28.5	95	37.5	49	19.4	21	8.3	16	6.3	253	3.74	1.147
38. Helps develop good relations amongst work group members.	79	31.1	86	33.9	43	16.9	23	9.1	23	9.1	254	3.69	1.252

Germain and Cummings (2010:433) suggested that nurses feel empowered when their nurse leaders show confidence in their abilities to perform. They furthermore stated that when nurses feel important by being able to make decisions, without seeking approval from their leaders, they feel empowered to perform. This produces job satisfaction, cost-effectiveness, better solutions and constructive conflict management. A number of researchers proposed that to manage changes and challenges in the healthcare system, nurse managers must move away from the traditional management paradigm of hierarchal power and control to a model of leadership that shares power and control (Bester et al. 2015; De Klerk & Stander 2014; Tomey 2009; Zhang & Bartol 2010).

### Relationship between resilience and empowering leader behaviour

Hoteling’s *t*-test showed a statistically significant relationship, where nurse managers with high resilience levels were perceived by team members to have statistically higher empowering leadership scores than managers with low resilience levels.

## Limitations of the study

The study was limited to one mining healthcare sector. The results of the study can thus not be generalised.

Data collection involved only the respondents’ self-completion of questionnaires. In-depth individual and focus group discussions with nurse managers and with the nurses of different categories could have produced more in-depth information.

## Recommendations

### Recommendations for nursing practice

Nurse managers should attend workshops and in-service training on strengthening resilience to handle adverse working conditions in the mining healthcare sector. Nurse managers must attend leadership development programmes, specifically leadership coaching, to prepare managers for coping with healthcare challenges. Factors such as informing and leading by example have to be included in leadership development programmes.

### Recommendation for future research

Research should be conducted to further explore the relationship between resilience and empowering leader behaviour of nurse managers in the mining healthcare sector of South Africa. Future studies should consider qualitative methods.

## Conclusion

It is clear that higher leadership qualities are related to higher resilience levels. The value of this study is that it has given attention to the under-investigated issue of resilience and empowering leadership behaviour in South Africa’s mining healthcare sector.
